# FLOWSA: A Python Package Attributing Resource Use, Waste, Emissions, and Other Flows to Industries

**DOI:** 10.3390/app12115742

**Published:** 2022-06-05

**Authors:** Catherine Birney, Ben Young, Mo Li, Melissa Conner, Jacob Specht, Wesley W. Ingwersen

**Affiliations:** 1 U.S. Environmental Protection Agency, Office of Research and Development, Center for Environmental Solutions and Emergency Response, Cincinnati, OH 45268, USA; 2 Eastern Research Group, Inc., Lexington, MA 02421, USA; 3 General Dynamics Information Technology Inc., Fairfax, VA 22042, USA; 4 Global Quality Corp., Edgewood, KY 41017, USA

**Keywords:** material flows, resource use by industry, water use by industry, waste by industry, emissions by industry, tool ecosystem, NAICS, sector allocation, Python, USEEIO, satellite accounts, physical accounts, national environmental accounts

## Abstract

Quantifying industry consumption or production of resources, wastes, emissions, and losses—collectively called flows—is a complex and evolving process. The attribution of flows to industries often requires allocating multiple data sources that span spatial and temporal scopes and contain varied levels of aggregation. Once calculated, datasets can quickly become outdated with new releases of source data. The US Environmental Protection Agency (USEPA) developed the open-source Flow Sector Attribution (FLOWSA) Python package to address the challenges surrounding attributing flows to US industrial and final-use sectors. Models capture flows drawn from or released to the environment by sectors, as well as flow transfers between sectors. Data on flow use and generation by source-defined activities are imported from providers and transformed into standardized tables but are otherwise numerically unchanged in preparation for modeling. FLOWSA sector attribution models allocate primary data sources to industries using secondary data sources and file mapping activities to sectors. Users can modify methodological, spatial, and temporal parameters to explore and compare the impact of sector attribution methodological changes on model results. The standardized data outputs from these models are used as the environmental data inputs into the latest version of USEPA’s US Environmentally Extended Input–Output (USEEIO) models, life cycle models of US goods and services for ~400 categories. This communication demonstrates FLOWSA’s capability by describing how to build models and providing select model results for US industry use of water, land, and employment. FLOWSA is available on GitHub, and many of the data outputs are available on the USEPA’s Data Commons.

## 1. Introduction

Attributing the consumption or generation of environmental and economic data to industrial and final-use sectors is integral to understanding the transactional relationship between the environment and the economy. Sectors are typically defined as economic sectors generating economic activity but are extended here to include household and government end-users. Sector attribution models capture the movements of resources (environmental, monetary, and human), wastes, losses, or emissions between the environment and sectors. The physical movements of material or energy can be generically called flows, a term adopted from life cycle assessment modeling [1]. Sector attribution models calculate direct resource use and emissions by sectors. Model results can be integrated into economic and environmental research applications, such as life cycle assessment (LCA) modeling, to quantify the embedded environmental impacts of goods and services [2].

In 2017, the US Environmental Protection Agency (USEPA) released the US Environmentally Extended Input–Output (USEEIO) models, a set of spreadsheet-based Excel^®^ models evaluating the environmental impacts of the US economy [2]. As with other Input–Output (IO)-based models [3,4], attributing the direct consumption or production of flows to sectors is incorporated within the models rather than being a standalone modeling process. Embedding sector attribution methods into other modeling objectives can lead to methodology limitations. The spreadsheet-based USEEIO models had three notable limitations. Aspects of modeling efforts were duplicated across the generation of environmental satellite tables because the attribution of flows to sectors for many environmental flows required the same methodological steps and overlapping data sources. Flow data were assigned to Bureau of Economic Analysis (BEA) industry codes; however, the BEA industry accounts definitions change over time, and data can be more accurately attributed to the more specific North American Industrial Classification System (NAICS) codes. Additionally, the USEEIO models attributed all flows to industries, whereas commodity attribution is more appropriate at times. The USEPA developed the Flow Sector Attribution (FLOWSA) Python tool to address these methodology limitations with sector attribution modeling.

FLOWSA is a publicly available data processing library designed to attribute flows of resources, wastes, emissions, and losses to sectors [5]. FLOWSA generally allocates flows to NAICS codes but can also attribute flows to household and government end-users, as well as user-defined sectors. Allocation is expanded beyond official NAICS, as NAICS represents US economic industries, but there is a need to track flows associated with non-industries, such as households. FLOWSA differentiates between commodity and industry flows, where a commodity is a good or service and an industry is an economic activity that produces commodities. FLOWSA aggregates, combines, and prepares data from publicly available sources to generate attribution models for various flow types, outputting results in standard tabular formats. Users can access or tailor existing flow models or create new models. Model methods can be modified to quantify sector flows for varying temporal and spatial scales, resource inputs, and data sources. Users can define the sector aggregation level (2- to 6-digit NAICS) based on data needs.

FLOWSA outputs two types of data: Flow-By-Activity (FBA) and Flow-By-Sector (FBS). FBA datasets are imported flow data that are formatted but numerically unchanged from the source and retain original activity names (e.g., “Irrigation Crop” or “Corn”). Dependent on the source data, activities are classified as either industries or commodities. FBA models transform source data, preserving relevant information for sector attribution modeling. Data are formatted into a standardized data structure because, regardless of the flow type, relevant sector-related data encapsulate the same information. The strict formatting of environmental and economic data imports streamlines the FBS dataset generation.

FBS datasets are environmental and economic data attributed to sectors. These datasets were developed out of a need for a standard output format that can capture the result of the sector attribution modeling and be useful for downstream uses. FBS datasets are useful for environmental and economic data-driven applications, such as for environmental data inputs into the USEEIO modeling efforts [6]. Typically, sector attribution models have been limited to capturing flows from the environment to sectors (resource input from the biosphere) and from sectors to the environment (emission to the biosphere). However, FLOWSA models can account for flows between sectors (within the technosphere).

**Figure 1.**
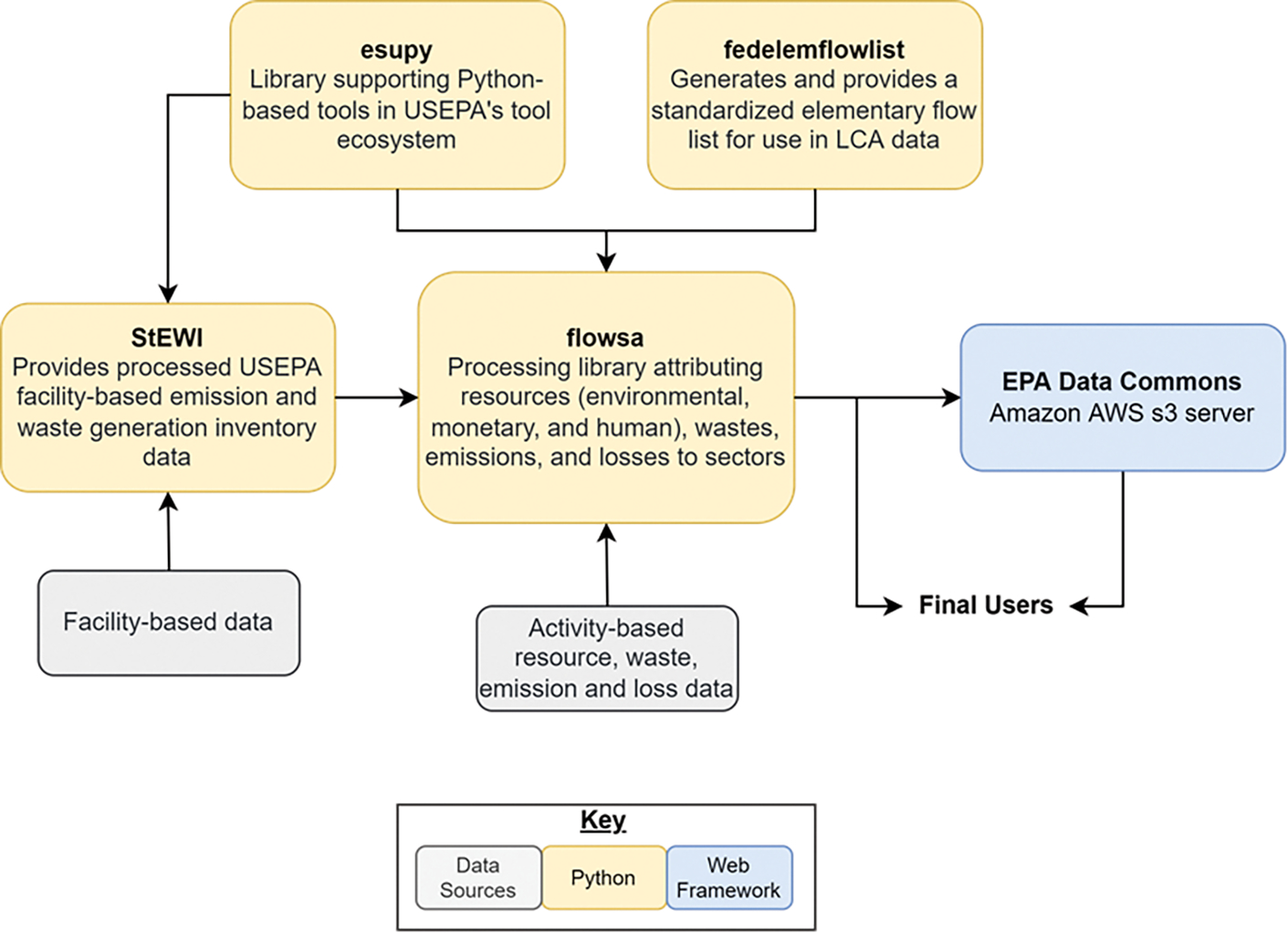
Schematic of the connections between FLOWSA and other USEPA life cycle assessment (LCA) ecosystem tools.

FLOWSA is a tool within the USEPA LCA tool ecosystem, a set of tools developed to support industrial ecology modeling [7]. FLOWSA relies on three additional USEPA Python packages to generate FBA and FBS datasets; the relationships between the packages are depicted in Figure 1. USEPA facility-level data are imported into FLOWSA from the Standardized Emission and Waste Inventories (StEWI) package. StEWI is a series of four Python packages that processes emission and waste generation inventory data for US facilities in standard formats [8]. FLOWSA reformats, aggregates, and assigns StEWI’s facility-level data to sectors. FLOWSA imports functions from esupy, a package that hosts generic functions used across all LCA tool ecosystem Python packages, and fedelemflowlist, a package developed to standardize elementary flows [1]. Datasets produced in FLOWSA are accessible to end-users in the Python package and on Data Commons, an Amazon AWS s3 server to which the USEPA uploads the most recent model results.

To the authors’ knowledge, FLOWSA is the first tool designed to advance the understanding and documentation of the direct use and emissions of environmental and economic data within the US economy and enable timely updates when new source data are published. FLOWSA streamlines the process of attributing flow data to sectors by using flexible functions across flow models, built-in data validation checks, and metadata generation. FLOWSA is designed to efficiently explore multiple methods of sector attribution for a single flow type, which will save researchers time. Although the models currently focus on the US, the package can be adapted for sector attribution modeling of non-US regions. FLOWSA is hosted on GitHub (https://github.com/USEPA/flowsa, accessed on 2 May 2022), enabling continuous improvement to the methodology and ensuring users can always access the most up-to-date code.

This paper demonstrates FLOWSA’s flexibility in generating sector attribution models for resource management and comprehensively introduces the tool. This paper includes detailed information on available data, models, validation, and data storage. The latest FLOWSA release, FLOWSA v1.2.1, is used to generate select model results.

## 2. Materials and Methods

**Figure 2.**
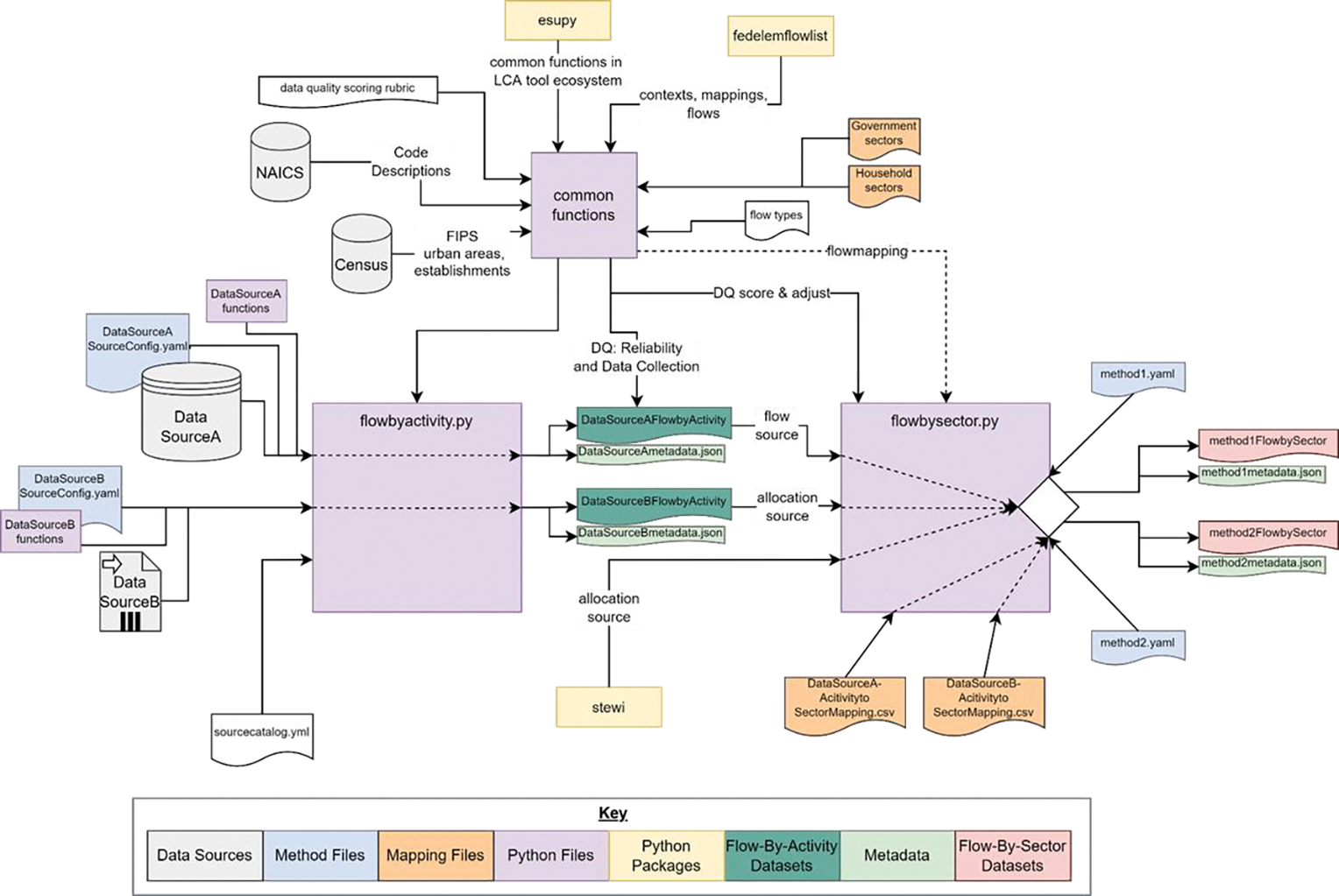
Schematic of the process to import and format source data as Flow-by-Activity (FBA) datasets, which are used to generate Flow-By-Sector (FBS) datasets.

This section highlights FLOWSA’s three primary data outputs and provides an overview of generating sector attribution graphics. A complete guide to individual functions is in the package README.md files and the GitHub wiki (https://github.com/USEPA/flowsa/wiki, accessed on 2 May 2022). The functionality of FLOWSA can be summarized as follows and is depicted in Figure 2:

1. Retrieves and formats publicly available environmental and economic data into Flow-By-Activity (FBA) tables;
2. Maps unique source activity names to sectors, generally NAICS codes, and classifies each sector as an industry or commodity;
3. Attributes source activities in FBA datasets to all related sectors using specified allocation methods, formatted into Flow-by-Sector (FBS) tables;
4. Enables model result exploration and comparison with data visualization functions.

FLOWSA is designed for use by both non-expert and expert users. Non-expert users can access existing model results on the USEPA Data Commons (https://edap-ord-data-commons.s3.amazonaws.com/index.html?prefix=flowsa/, accessed on 2 May 2022) server without using Python, or they can install FLOWSA and run simple commands to load datasets. Expert users can clone the GitHub repository and use FLOWSA as a developer to create user-customized FBA or FBS datasets. Developers can modify the existing FBA and FBS methodologies or create new models.

### 2.1. Flow-by-Activity (FBA) Datasets

The first step in the sector attribution modeling process is importing and formatting publicly available data into standardized FBA tables while maintaining names, quantities, and units. FBA tables capture the physical exchanges between source activities and the environment or between two activities. Activities are industries or commodities (e.g., “Manufacturing” or “Cows”) producing or consuming a flow (e.g., “CO2” or “Water”). The activity names in an FBA dataset are unchanged from those in the source data. Data are organized by assigning activity names to either the “ActivityProducedBy” or “ActivityConsumedBy” column, depending on whether the activity produces or consumes a flow. Both activity columns are filled when one activity consumes a flow produced by a second activity, such as when “Domestic” users consume water withdrawals produced by “Public Supply”. The unmodified source data can be repeatedly called across sector attribution models for use as primary or allocation data sources, depending on the need for the data. Generalized functions import data from websites, APIs, PDFs, and CSV files, usually accessed through a URL request.

All FBA tables are formatted with the same column headings and structured to capture information specific to sector attribution modeling. The table specifications are located in the Supplementary Information (SI) file and the “format specs” directory in the FLOWSA GitHub repository. Most columns are subset source data, such as the original activity names, flow values, units, and uncertainty values. The FBA tables also include data helpful for sector attribution modeling but not specified in the source. This information includes the data source name, which follows a standardized naming convention specific to the FLOWSA package, compartment, flow type, and flow class.

All flow information are assigned to standardized data columns. Compartments capture where the flow is found, e.g., “air”, “water”, or “ground.” Flow types capture how the flow moves, classified as elementary, technosphere, or waste flows. Elementary flows are flows drawn from or released to the environment without human transformation, while technosphere flows are intermediate flows [1]. Waste flows capture the end of life of a flow. Flow classes are the classification of data included in the FBA, such as “Chemicals” or “Energy”. The flow class assignments follow the Federal Life Cycle Assessment (LCA) Commons Elementary Flow List. The fedelemflowlist package is a Python package developed to support the Federal LCA Commons by producing standardized elementary flows for life cycle assessment data [9]. Flows are standardized by assigning unique flow IDs to combinations of compartments, flow types, and flow classes, regardless of the terminology used in the source data. The flow classes and flow types identified in FLOWSA v1.2.1 are listed in Table 1. The FBA tables also record data quality scores for data reliability and collection, calculated using a standardized system developed by Edelen et al. [10].

Each time an FBA dataset is created, the data and a metadata file are stored in a user’s local directory. The metadata file timestamps the data access and captures information specific to FLOWSA at the time of the FBA generation, such as the package version number and current git hash. In FLOWSA v1.2.1, 51 unique data sources generate 450 Flow-By-Activity datasets, as listed in Table 2. Users can access a current list of available FBA models using the following function: flowsa.seeAvailableFlowByModels (‘FBA’).

### Flow-by-Activity Generation

Many functions in FLOWSA are generic and can be used to import and format data regardless of the source. These functions range in purpose from calling URLs to processing data frames. Although each FBA dataset requires functions unique to the data source, much of the process of generating a new FBA is automated, as depicted in Figure 2. In addition to the generalized scripts, FBA generation requires two files specific to the imported data source. The first is a human-readable YAML configuration file that acts as instructions by housing parameters to locate the data and any specific function names required to generate the FBA. The second is a Python file with functions to help pull, parse, and format the data into the standardized FBA columns. These functions are listed in and loaded from the configuration YAML. Running the script “flowbyactivity.py” generates the Flow-By-Activity dataset by loading the YAML configuration file and reading the instruction-like parameters. The YAML method file is easily updatable when new source data is published, as the method file often only requires the additional years or a dictionary of new column names. To retrieve an FBA as a pandas data frame [11], a user can call on the customizable “getFlowByActivity” function. To generate FBA datasets not currently included in FLOWSA, users can create a customized configuration file in the format of the built-in method YAML. flowsa.getFlowByActivity (datasource, year, flowclass = None, geographic_level = None, download_FBA_if_missing = False).

The data source and year parameters must be an available combination of source data and source data year. The FBA can be subset to return a specific type of flow class or a geographic scale, or, if left to the function defaults, “None”, the retrieved dataset will contain information for all available flows and geographic scales. The final function option, “download_FBA_if_missing”, indicates that if the FBA is not found in a user’s local directory, the FBA should be downloaded from USEPA’s Data Commons.

## 2.2. Mapping Flow-by-Activity Datasets to Sectors

The next step of the sector attribution process is determining which sectors relate to each activity in the FBA datasets. Generating an FBS requires each FBA to have a unique concordance file containing the source activity names, such as “Irrigation Crop”, matched to one or more related sectors. Sectors are generally 2012 NAICS codes but also include the BEA codes for household and government sectors, as no NAICS codes represent these sectors. The only data sources that do not require a mapping file are those with activities that are already published as NAICS. Activities are mapped to the most aggregate, appropriate NAICS level. NAICS codes are published in a two- to six-digit hierarchy, where the economic sector becomes more specific as the digits increase. The activity-to-sector mapping or “crosswalk” files capture which sectors are related to an activity but do not specify how activities are attributed to sectors. Rather, the FBS method dictates the attribution methodology. Sector assignments in FLOWSA v1.2.1 were created by conversing with data publishers and using source-provided concordance files, activity definitions in publications, and NAICS definitions [12].

As crosswalks are manually created for each data source, users can include customized sector codes to disaggregate an economic activity beyond the standard categorization. In FLOWSA v1.2.1, many of the activities from the US Department of Agriculture (USDA) Census of Agriculture (CoA) [13] are assigned to 7-digit NAICS codes. For example, NAICS defines sector code 112130 as “Dual-Purpose Cattle Ranching and Farming” [12], but the relevant data available from the USDA CoA is for (a) “Cattle, (Excl Cows)” and (b) “Cattle, Cows”. As the USDA data are more specific than the definition of the 6-digit NAICS code and because the two values combined equal the 6-digit NAICS, “Cattle, (Excl Cows)” is assigned a sector code of “112130A” and “Cattle, Cows” a sector code of “112130B”. This flexibility in sector assignments allows the flow activity to be imported without modification while accurately attributing the data to sectors.

A subset of the USGS National Water Information System (NWIS) Water Use (WU) [14] crosswalk is shown in Table 3, with the standard mapping table headers. The “ActivitySourceName” column is the acronym of the data source name used throughout FLOWSA and is unique to each data source. The “Activity” column contains original activity names from a source mapped to sectors. The “SectorSourceName” specifies the year of the NAICS codes used. In FLOWSA v1.2.1, all FBA mapping files use 2012 codes. The “Sector” column contains the most aggregate applicable NAICS for each source activity. The column “SectorDescription” is added here to clarify the sector codes but is not included in the FLOWSA code.

In the Industrial activity subset, water withdrawals are mapped to multiple NAICS, ranging between 2-digit and 6-digit codes. Each mapping indicates that an activity is related to all child NAICS of a parent NAICS, so an activity mapped to a 2-digit NAICS is also attributed to all 6-digit child NAICS. For example, Industrial water withdrawal maps to all child NAICS of Construction (“23”) but only maps to Logging (1133), not to any of the parent NAICS within Agriculture, Forestry, Fishing, and Hunting (11). Water withdrawals related to other agricultural sectors are captured in a separate USGS activity. The final column included in the mapping file is “SectorType”, where each sector is assigned an industry (“I”) or commodity (“C”) association.

## 2.3. Flow-by-Sector Datasets

The standardized output from sector attribution models is a flow-by-sector (FBS) dataset derived from the FBA datasets by applying an FBS method. The method identifies FBA datasets required to generate the FBS dataset, where FBA data can be used as either primary flows or for allocation. The primary FBA data are subset by activities and attribution methods, such as “proportional” or “direct”. Each attribution method attributes the activity subsets to sectors using additional FBA data identified as allocation sources. For each FBA dataset, the FBS method identifies the required sector definition, geographic aggregation, and flow names. The FBS methods are flexible, with modifiable target sector levels, where a user can specify the desired sector aggregation level (2- to 6-digit NAICS) or a combination of sector levels.

Like FBAs, FBS data are output in a table with standardized headers. A complete table format description is found in the manuscript SI and FLOWSA’s format specs directory. The information in the FBS is transformed primary FBA data, with information about any allocation datasets captured in the “MetaSources” column and recorded in the metadata file generated with each FBS. FLOWSA transforms the primary FBA data in three ways. The first is by converting all units to the International System of Units. The second is mapping the FBA to the USEPA’s Federal LCA Commons Elementary Flow List [9], creating standardized Context and Flow UUID columns, following the Federal LCA Commons guidelines [1]. The Context column contains a text string indicating the directionality of the flow between the environment and sector, e.g., “emission/air/troposphere”, derived from the FBA’s compartment column. The Flow UUIDs are unique hexadecimal IDs for each flow name and context combination, harmonizing flow data regardless of source activity names. The third data transformation occurs for primary and allocation FBAs by mapping the source activities to sector codes. This mapping converts the “ActivityProducedBy” and “ActivityConsumedBy” columns to “SectorProducedBy” and “SectorConsumedBy”. Data in the primary dataset are allocated to sectors using the specified allocation method in the FBS method file. The FBS retains much of the primary FBA, such as the flow name, flow class, geographic location, and flow type. Future enhancements to FLOWSA will capture data quality scores for data reliability, temporal correlation, technical correlation, and data collection using the scoring framework developed by Edelen et al. [10].

FLOWSA v1.2.1 includes 25 FBS datasets. The methods and strings used to generate the FBS are listed in Table 4. It is possible to have multiple sector attribution methods for a flow type and location because models can be created using different allocation data or attribution methods.

Users can check an up-to-date list of available FBS and call on the “getFlowBySector” function to retrieve an FBS dataset. flowsa.seeAvailableFlowByModels (‘FBS’), flowsa.getFlowBySector (methodname, download_FBAs_if_missing = False, download_FBS_if_missing = False).

Where methodname is the name of the FBS, identified by either running the function to see the available models or, if using FLOWSA as a developer, an FBS created by a user. Users can download the FBAs required to generate the FBS from the USEPA’s Data Commons or download the FBS itself by changing the default values to “True” in the function. If the default download settings are set to the default “False”, FLOWSA will run any required scripts to create FBAs and the FBS.

Output model results have two sector columns, “SectorProducedBy” and “SectorConsumedBy”, that capture flows of data from one sector to another. FBS data that only contain elementary flows will include a blank sector column, representing that the flow is produced by or consumed by a sector, released to or withdrawn from the environment, respectively. This empty sector column can be dropped by calling on the following function to collapse the sector columns flowsa.collapse_FlowBySector (methodname, download_FBAs_if_missing = False, download_FBS_if_missing = False).

### Flow-by-Sector Generation

FBS models require (1) a YAML method file containing parameters loaded as instructions on how to attribute activities to sectors, (2) locally stored primary and allocation FBAs, and (3) crosswalk mapping source activity names to sector codes, as indicated in Figure 2. The FBS YAML method file hosts a human-readable dictionary of instruction-like parameters for attributing or allocating primary data to sectors using allocation FBA datasets. Subsets of the primary FBA activities are attributed to sectors by allocation FBAs and methods identified in the method file. The most common methods are “direct” and “proportional” allocations. Direct allocation does not require any allocation data to assign flow to sectors; the ratio is 1:1. Proportional allocation requires at least one additional data set to create ratios for data allocation because an activity cannot be directly mapped to sectors when an activity is mapped to multiple sectors. Additional data allocation methods in FLOWSA v1.2.1 include “scaled”, “multiplication”, “weighted average”, and “disaggregation”. Descriptions of each allocation method are included in the FBS methods README.

Like the FBA generation models, FBS functions are generically written to enable use across all sector attribution models, regardless of the flow. Individual functions load FBAs, map data, convert units, estimate suppressed data, and allocate data using the FBA and FBS table structures. FBS allocation often requires specific functions for primary or allocation data sources to help allocate an FBA dataset to sectors. These helper functions are housed in the same Python file used to generate the Flow-By-Activity, organized by the data source name. These functions are optional and dependent on the data source. FBS datasets are generated by running “flowbysector.py”, a script that calls on the information and functions specified in the Flow-By-Sector methods YAML. Users can create their own FBS method files for flows not currently included in FLOWSA or modify existing FBS method files or crosswalks to meet data needs.

Generating an FBS dataset relies on additional USEPA industrial ecology ecosystem tools [7], as depicted in Figure 1. In addition to all primary FBA flows mapped to the Federal LCA Commons Elementary Flow List, some functions used in FLOWSA are imported from esupy, a package that houses common functions across several USEPA Python-based ecosystem tools [15]. Several FBS datasets rely on data imported from the Standardized Emission and Waste Inventories (StEWI) Python package [16], which processes USEPA facility-based emission and waste generation inventory data. These datasets include the Toxic Release Inventory (TRI) [17], National Emissions Inventory (NEI) [18], Discharge Monitoring Reports (DMRs) [19], and Resource and Conservation Recovery Act Biennial Hazardous Waste Reports (RCRAInfo) [20]. Once imported, FLOWSA further processes the facility-based data by assigning data to sectors. FLOWSA also maps the data using the Federal LCA Commons Elementary Flow List [9], filters and cleans data for the FBS (e.g., adjusts for airplane emissions or removes GHGs from NEI data), and assigns and aggregates geographic locations.

## 2.4. Data Visualization Functions

One of the objectives of FLOWSA is to provide a modeling platform where model methodology can be easily modified and compared to other methods. FLOWSA includes a function for model results visualization to (1) assist in determining the impact of methodological variation or (2) understand the direct flows attributed to sectors. The function below produces both plots flowsa.generateFBSplot (method_dict, plottype, sector_length_display = None, sectors_to_include = None, plot_title = None).

Where method_dict is a dictionary of data to include in the plot, the dictionary key is the data title, and the dictionary value is the FBS method. The plottype is either “facet_graph”, which compares multiple flow types for a subset of sectors, or “method_comparison”, which plots model results for different flow methodologies on the same plot. Users can specify the NAICS sector length to display, a subset of sectors to include in the plot, and a graph title.

## 3. Results

FLOWSA v1.2.1 was developed with 13 collaborators, consisting of over 24,000 lines of Python code split into 109 modules, supported with 136 YAML and 78 CSV files. Results of a code profile of FLOWSA v1.2.1 generated with the Statistic IntelliJ IDE plugin [21] are presented in Table 5. FLOWSA v1.0 underwent an internal USEPA peer-review process; FLOWSA v1.2.1 includes code updates since the review. The USEPA will continue to maintain and enhance FLOWSA’s capability as the program is an integral component of the USEEIO family of models [6]. Future versions of FLOWSA will undergo additional internal USEPA peer reviews when there are significant package modifications.

### 3.1. Conceptual Water Withdrawal Flow-by-Sector Methodology Example

This section conceptually walks through an example national-level water withdrawal sector attribution model in FLOWSA. The primary data source for a water withdrawal FBS is USGS Water Data for the Nation, which contains national, state, and county water withdrawal information for nine broad water use categories or activities, including “Irrigation Golf Course”, “Livestock”, and “Mining” [14]. The USGS data are imported to FLOWSA and output as a formatted FBA for sector attribution modeling. The activity-to-sector mappings is created using NAICS definitions [22] and a concordance file provided by the USGS. The objective of the water FBS is to attribute the USGS data to national-level 6-digit sectors, requiring multiple allocation methods and FBAs. The “Irrigation Golf Course” activity can be directly assigned to the 6-digit NAICS “713910”, Golf Courses and Country Clubs. In contrast, the activity “Livestock” can only be directly mapped to the 3-digit NAICS “112”, Animal Production and Aquaculture, and requires additional data sources to allocate to 6-digit NAICS. The Livestock water withdrawal cannot be directly allocated to 6-digit NAICS with the USGS data alone because it is unclear how much water is used by Beef Cattle Ranching and Farming (112111) versus any other animal. A proportional allocation method is required to allocate “Livestock” water withdrawal accurately to different animal types. The first step is to use data on the number of animals in each animal type category [13] and multiply that by estimates of drinking water requirements by animal type [23]. The result is a value of total annual drinking water by animal type. The USGS 3-digit NAICS “Livestock” water withdrawal is proportionally allocated to 6-digits using the calculated water use by animal type. The calculated water use by animal types is not used directly because the water FBS represents water for all NAICS. Using the USGS as the sole primary data source ensures that the water allocation method accounts cumulatively for all published water withdrawals in the US. Other USGS water data activity subsets, such as “Mining”, require different allocation data sources and methods to allocate water withdrawal to 6-digit NAICS. Users can modify each water withdrawal category’s data sources and allocation method within the FBS method YAML.

## 3.2. Select Flow-by-Sector Model Results

**Figure 3.**
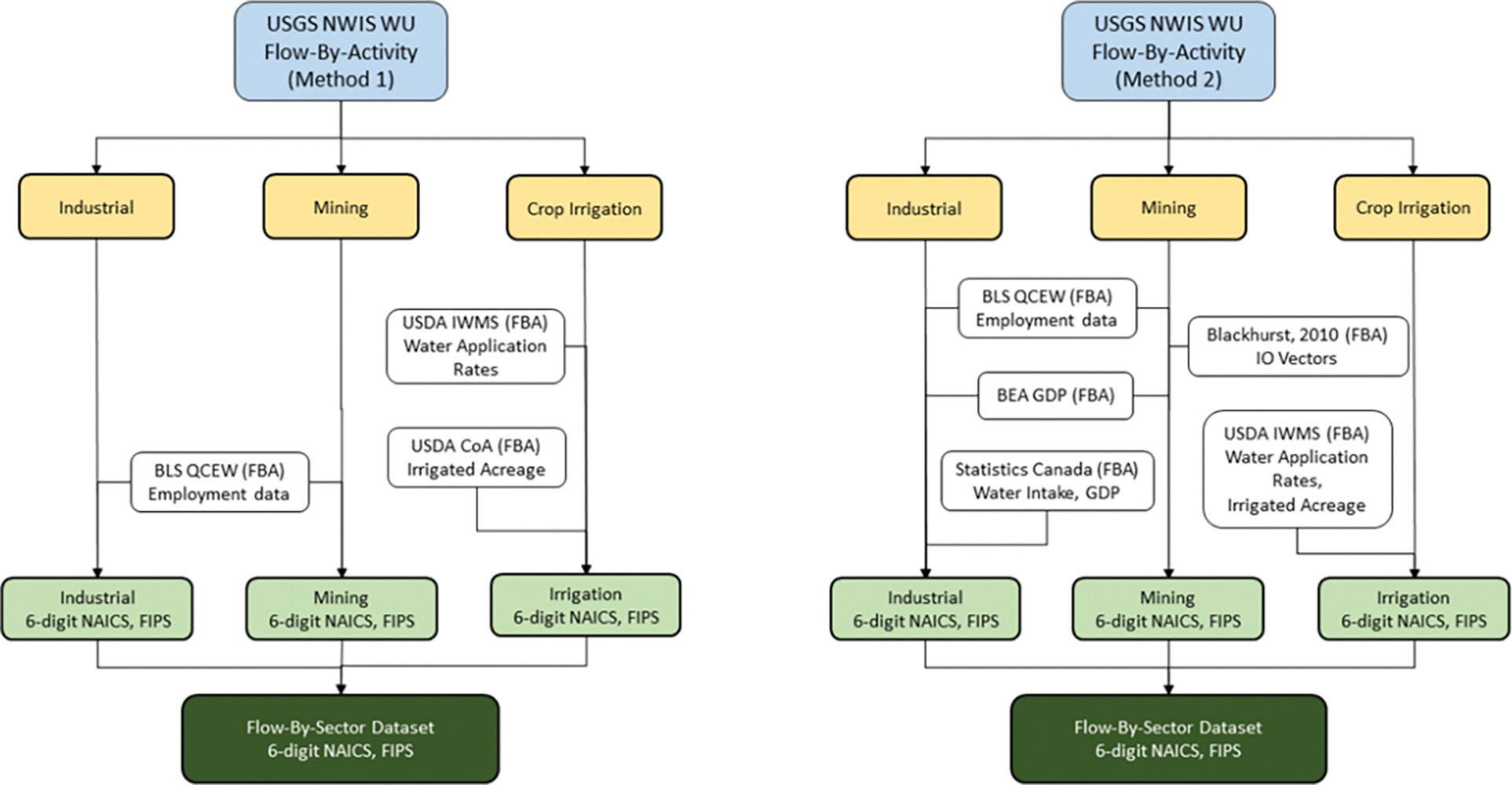
Comparison of the data sources used to generate the Method 1 and Method 2 National Water Withdrawal Flow-By-Sector datasets. FBA is defined as “Flow-By-Activity”. Data Sources: USGS NWIS WU, BLS QCEW, USDA IWMS, USDA CoA, BEA GDP, Statistics Canada, and Blackhurst IO Vectors.

The FLOWSA v1.2.1 release contains three methods for attributing the national 2015 USGS water withdrawal to 6-digit NAICS. Figure 3 depicts the difference in allocation datasets used for Method 1 and Method 2. Of the nine USGS water-use categories, the allocation methods are modified for Industrial, Mining, and Crop Irrigation. The two methods use different data sources [13,14,24–27] with varied temporal and spatial scales. These methodological differences are captured in the human-readable YAML method files. Multiple water withdrawal methodologies enable a comparison of the impact of different methods on industry attribution.

**Figure 4.**
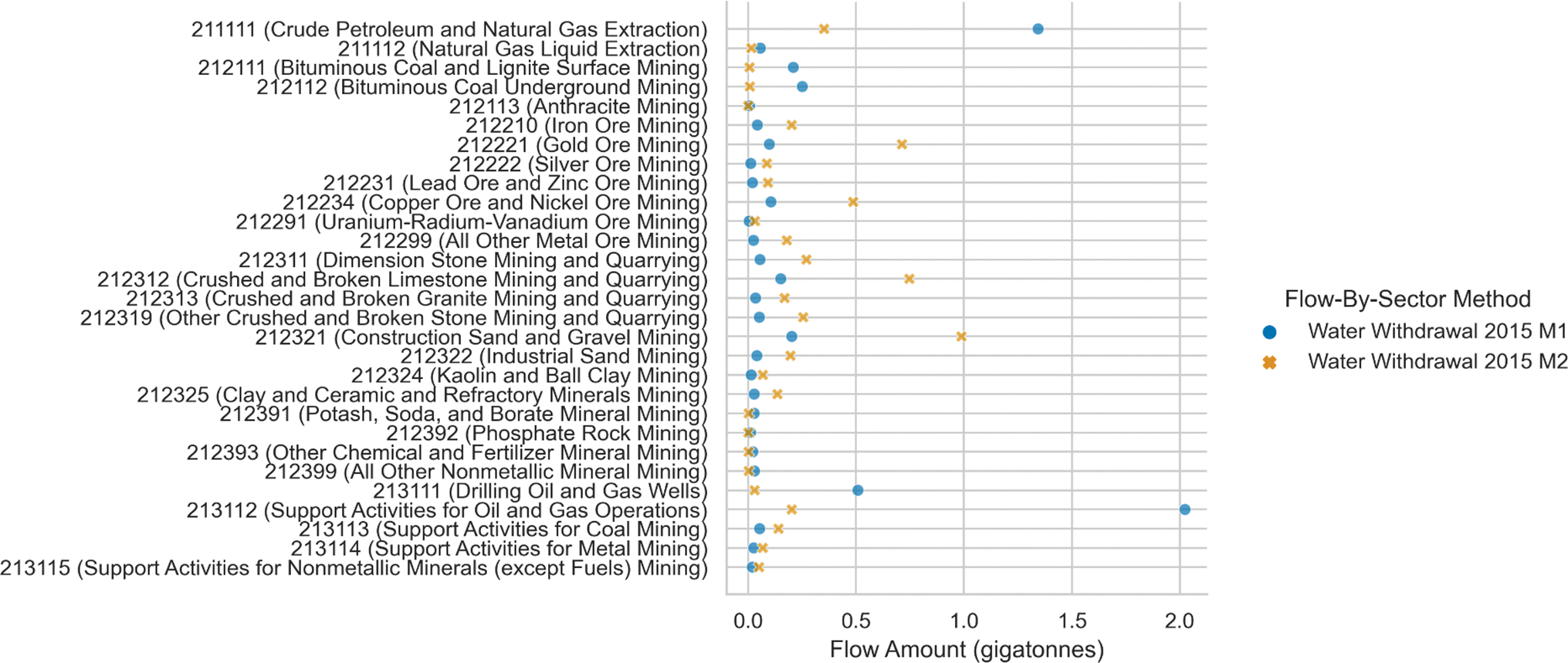
Comparing direct water withdrawals by 6-digit NAICS in 2015. Method 1 and Method 2 results were generated using the FBS methods “Water_national_2015_m1” and “Water_national_2015_m2,” respectively, with FLOWSA v1.2.1.

Figure 4 shows the difference in water withdrawal model outputs at the 6-digit NAICS between water withdrawals Method 1 and Method 2 for Mining, Quarrying, and Oil and Gas Extraction sectors (NAICS 21). Although total water withdrawal is the same for both methods, the water withdrawal rates by sector differ because the primary allocation source in Method 1 is BLS QCEW employment data [24], while Method 2 relies on 2002 IO vectors, as published by Blackhurst et al. [4]. As Method 2 attributes water withdrawals to 6-digit NAICS with data from 2002, the results are likely not a good representation of water withdrawals for mining activities in 2015 due to an increase in natural gas production [28]. Method 1 attributes more water to Crude Petroleum and Natural Gas Extraction and Support Activities for Oil and Gas Operations, capturing the increased natural gas extraction in 2015 compared to 2002.

**Figure 5.**
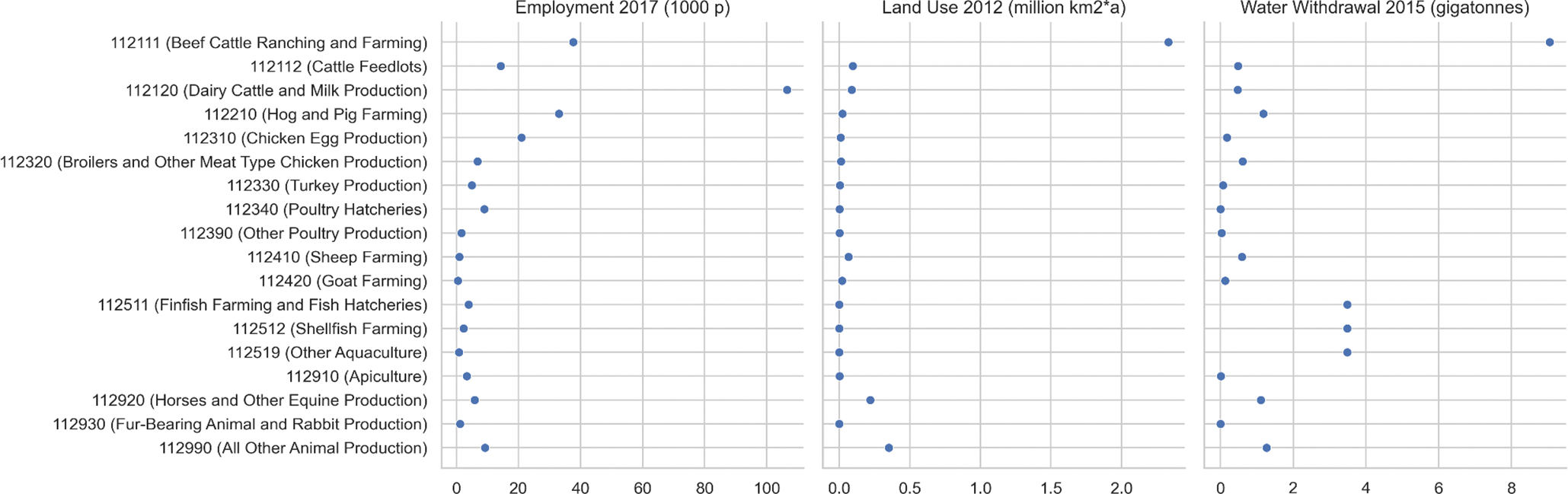
National employment, land use and water withdrawal for crops by 6-digit NAICS. The datasets were generated using the FBS methods “Water_national_2015_m1”, “Land_national_2012”, and “Employment_national_2016” with FLOWSA v1.2.1. The unit “p” for employment is persons. The unit “km2*a” for land use is square kilometers occupied per year.

By utilizing all environmental and resource data available in FLOWSA, users can determine the direct raw materials and human resources required for a subset of NAICS. Figure 5 represents the direct water withdrawal, land use, and employment required for Animal Production and Aquaculture (NAICS 112) in the United States. The water withdrawal in this figure includes water for animal consumption, irrigating pastureland, and aquaculture. Land use represents land used directly by animals, animal operations, and pastureland. The values in this figure exclude indirect flows, such as water withdrawal or land use for crops intended for animal feed. Capturing the indirect flows requires these direct resource use results to be input into an LCA model such as USEEIO [6].

## 4. Discussion

FLOWSA is an open-source modeling tool that generates standardized sector attribution tables for the direct use of environmental and economic data by sectors within the US. By hosting the package on GitHub, users can always access the most up-to-date code and model results. Due to GitHub’s built-in version control, users will retain data access and model reproducibility. Storing model results on USEPA’s Data Commons allows end-users and software to access the data without installing Python. FLOWSA is designed to be flexible and allow for convenient and rapid exploration of the flows used or produced by the US.

### 4.1. Sector Attribution Challenges

FLOWSA is designed to overcome common data-, modeling-, and platform-related challenges. Methods attributing flows to sectors face data processing challenges, regardless of the flow type. Problem-solving functions in FLOWSA are often written generically, relying on data frame structure rather than flow type to allow the use of the same functions across all sector attribution methods. There are two types of common sector attribution challenges: (1) dataset-specific obstacles and (2) disparities between primary and allocation data.

The main dataset-specific obstacles surround missing data at the target sector level. Many datasets contain suppressed data to protect the identity of individuals or organizations. To prevent data loss caused by missing data when alternative datasets can not be used to fill in the data gaps, FLOWSA includes a function to estimate the suppressed data. Data is estimated by equally allocating a parent sector within a location to all suppressed child sectors, accounting for any published child sector values. Additionally, at times, an activity can only be mapped accurately to a particular sector level, and there are no allocation datasets to further disaggregate data to a target sector level. In this situation, a function is called to allocate the data equally to all child sectors from the known sector level. Future versions of FLOWSA will likely incorporate additional methods of data suppression estimation.

Before merging datasets for allocation purposes, data discrepancies must often be addressed, including harmonizing units, Federal Information Processing System (FIPS) codes, temporal scales, and spatial scales. Some challenges are quickly addressed, such as differences in units or FIPS assignments by data year. FLOWSA contains a function to convert all data to the International System of Units upon loading an FBA for use in an FBS method. Over the years, county-level FIPS codes have changed due to the redrawing of country districts. FLOWSA has a concordance file mapping FIPS codes over time and a function that assigns the correct county code based on the data year. Other data disparities, such as temporal or spatial disparities, are addressed by user-defined methodology or rules embedded in the model code. All methods in FLOWSA v1.2.1 allocate primary FBA data using the closest available year of data for allocation datasets. Users can modify this methodology by changing the FBS method file. One frequent restriction when combining datasets is differences in spatial scales between FBAs. Generally, spatial data are represented by US 5-digit FIPS codes representing the county, state, or national data. Non-US data is identified using International Organization for Standardization (ISO) country codes. Synthesizing spatial scales is automated in FLOWSA, with rules dependent on the spatial differences. When merging two data frames, the less aggregated spatial data are aggregated to the higher level of geographic data. A greater geographic scale is never disaggregated to a lesser geographic scale. At other times, if specified in the allocation method, a greater geographic scale represents data for a less aggregated geographic level, such as using national-level data to allocate state-level data.

In addition to data challenges, FLOWSA has built-in capabilities to address common modeling challenges. Data validation functions ensure that the model results are accurate and that data are not lost after manipulating the source data. The Flow-By-Sector datasets are validated with numeric checks run during dataset generation. When allocating source data activities to sectors, flows are checked for data loss by comparing source data values to the final Flow-By-Sector flow amounts. Data loss is generally less than 0.5%, except for cases of intentional data removal to avoid double counting. Additional checks include summing allocation ratios at each sector level to ensure no significant data differences between the different sector code lengths or data loss after allocation. A geographic data check is included when a source has multiple geographic scales, comparing published national values to summed lesser geographic scales. Child sectors are summed to parent sectors to ensure there is no data loss between sector levels. Validation results are output in a log file saved to a user’s local directory.

The objective behind the FLOWSA design is to create transparent and reproducible results in an open-source and collaborative environment and to overcome platform-related challenges that many models face. FLOWSA is developed in Python to prevent performance limitations that other platforms can face due to the large data size. The code is hosted on GitHub, enabling convenient and timely source data updates and sector attribution models. FLOWSA is designed to ensure version control and model reproducibility so users can always access the model code and data later. Github is an open-source environment and a means of version control, as each code update is captured with a unique git hash, ensuring model reproducibility. To further ensure model reproducibility, each time an FBA or FBS is generated, FLOWSA creates a metadata file, saved to a user’s local directory, that timestamps and captures the location of source data retrieval. JSON files capturing metadata are created when a Flow-By-Activity or Flow-By-Sector dataset is generated. A Flow-By-Activity metafile records the date the dataset is generated, bibliographic information on the dataset, and a link to the GitHub methodology at the time of running. A metafile for a Flow-By-Sector dataset is a compilation of all Flow-By-Activity metafiles and the bibliographic information for additional information, such as values taken from the literature and used in calculations. Both metadata files record the FLOWSA package version and git hash at the time of a model run. Capturing metadata is a way to record changes in upstream data files. An FBS can have many underlying datasets, changing over time as new source data is published or errors are corrected. It is essential to know what version of each FBA was used to construct each FBS.

An FBS metadata file might show that the FBS was generated using FBAs with various FLOWSA package versions and git hashes. New version releases of the package do not necessarily mean FBA datasets have changed. New releases capture that some code has changed or was added. The metafiles might reflect that an FBS was generated with older versions of FBAs.

### 4.2. FLOWSA Integration with Life Cycle Assessment Modeling

FLOWSA’s FBS datasets are used as model inputs in useeior, a publicly available R package [29] for building the US Environmentally Extended Input–Output (USEEIO) models [6]. The USEEIO models calculate the life cycle environmental and economic impacts of producing or consuming goods and services in the United States [30]. The use of FLOWSA datasets allows for timely updates to the useeior models when new environmental data is released.

### 4.3. Potential Applications of FLOWSA

The FBA and FBS datasets included in v1.2.1 focus on US-based environmental and economic data. However, FLOWSA can be expanded to import data from other countries, as FBA and FBS data structures require fields for “Location” (e.g., “00000”, the national-level US FIPS code) and “LocationSystem” (e.g., “FIPS_2015”, the FIPS codes from 2015). FLOWSA v1.2.1 generates FBA tables for Statistics Canada data [27], where the location system is assigned the ISO code for Canada. FLOWSA can be adapted to import and process data for additional countries. Sector attribution modeling for non-US regions could potentially benefit LCA consumption-based models that want to capture the impacts from outside the US.

FLOWSA outputs can be useful for many environmental or economic data-driven applications. Any dataset related to economic sectors can be imported and mapped to related sectors. FLOWSA is especially beneficial for LCA and IO applications. As previously discussed, the outputs of FLOWSA are currently used as inputs to the US Environmentally Extended Input–Output model. FLOWSA could be used to prepare data for standardized environmental–economic accounts for the US, which also require determining flows of environmental data associated with economic sectors [31].

### 4.4. Future Work

Future releases of the FLOWSA package will include additional flow models for new flow types. Planned sector attribution models include greenhouse gas emissions, commercial non-hazardous waste, nitrogen and phosphorus releases from agriculture, pesticide releases, mineral extraction, and energy extraction. Additionally, future releases will expand on state-level models for current and planned flow-by-sector models.

FLOWSA’s FBAs include data quality scoring for data reliability and collection based on a qualitative assessment created by the USEPA [10]. Data quality assessment will be expanded to assess temporal, spatial, and technological correlation scores in the FBS. When combined with methodological variation, data quality scores provide insight into the trade-offs of the sector attribution methods.

## Supplementary Material

Supplementary Material

## Figures and Tables

**Table 1.** Flow classes present in FLOWSA v1.2.1.

Flow Class	Description	Flow Types
Chemicals	Emissions of chemicals and groups of chemicals	ELEMENTARY_FLOWS
Employment	Jobs	ELEMENTARY_FLOWS
Energy	Energy consumption, transfer as electricity or waste heat	All types
Geological	Mineral and metal use	All types
Land	Land area occupied	ELEMENTARY_FLOWS
Money	Purchases	TECHNOSPHERE_FLOWS
Water	Water use and release data, including wastewater	All types
Other	Misc flows used for supporting data	All types

**Table 2.** Flow-by-activity datasets present in FLOWSA v1.2.1.

Code	Dataset	Class	Geographic Scale	Description	Years
BEA_GDP_GrossOutput	Bureau of Economic Analysis Gross Output by Industry	Money	National	Gross output	2007–2018
BEA_Make_AR	Bureau of Economic Analysis Make Table After Redefinition	Money	National	Gross output, producer value, after redefinition	2002
BEA_Make_Detail_BeforeRedef	Bureau of Economic Analysis Make Before Redefinitions	Money	National	Gross output before redefinition, detail level	2012
BEA_Use_Detail_PRO_BeforeRedef	Bureau of Economic Analysis Use Before Redefinitions	Money	National	Gross output before redefinition, detail level, producer value	2012
BLM_PLS	Bureau of Land Management Public Land Statistics	Land	National	Land resources and information	2007, 2011, 2012
BLS_QCEW	Bureau of Labor Statistics Quarterly Census of Employment and Wages	Employment, Money, Other	National, State, County	Number of employees per industry, Annual payroll per industry, Number of establishments per industry	2002, 2010–2018
Blackhurst_IO	Input–Output Vector of 2002 Water Withdrawals for the United States	Water	National	Input–Output vectors of US water withdrawals	2002
CalRecycle_WasteCharacterization	CalRecycle	Other	California	Disposal-Facility-Based Characterization of Solid Waste in California	2014
Census_CBP	Census Bureau County Business Patterns	Employment, Money, Other	National, State, County	Number of employees per industry, Annual payroll per industry, Number of establishments per industry	2010–2017
Census_PEP_Population	Census Bureau Population Estimates	Other	National, State, County	Population	2010, 2013–2017
Census_VIP	Value of Construction Put in Place	Money	National	Construction Spending	2009–2020
EIA_CBECS_Land	Energy Information Administration Commercial Buildings Energy Consumption Survey	Land	National	Floorspace by building type	2012
EIA_CBECS_Water	Energy Information Administration Commercial Buildings Energy Consumption Survey	Water	Country	Water consumption in large buildings	2012
EIA_MECS_Energy	Energy Information Administration Manufacturing Energy Consumption Survey	Energy, Other	Region	Fuel and nonfuel consumption of energy flows by manufacturing industries	2010, 2014, 2018
EIA_MECS_Land	Energy Information Administration Manufacturing Energy Consumption Survey	Land	National, Regional	Floorspace by building type	2010, 2014, 2018
EIA_MER	Energy Information Administration Monthly Energy Review	Energy	National	Energy consumption and production	2010–2020
EPA_CDDPath	Construction and Demolition Debris	Other	National	Estimates of amount and disposition of Construction and Demolition materials	2014
EPA_EQUATES	Air QUAlity TimE Series Project	Chemicals		Chemical atmospheric concentrations and deposition	2002–2017
EPA_GHGI	Inventory of U.S. Greenhouse Gas Emissions and Sinks	Chemicals, Energy, Other	National	US GHG emissions and sinks by source, economic sector, and greenhouse gas	2010–2019
EPA_NEI_Nonpoint	Environmental Protection Agency National Emissions Inventory Nonpoint sources	Chemicals	County	Air emissions of criteria pollutants, criteria precursors, and hazardous air pollutants	2008, 2011, 2014, 2017
EPA_NEI_Nonroad	Environmental Protection Agency National Emissions Inventory Nonroad sources	Chemicals	County	Air emissions of criteria pollutants, criteria precursors, and hazardous air pollutants	2008, 2011, 2014, 2017
EPA_NEI_Onroad	Environmental Protection Agency National Emissions Inventory Onroad sources	Chemicals	County	Air emissions of criteria pollutants, criteria precursors, and hazardous air pollutants	2008, 2011, 2014, 2017
EPA_NI	Nitrogen Inventories	Chemicals	HUC8	Nitrogen inputs and fluxes	2002, 2007, 2012
EPA_PI	Phosphorus Inventories	Chemicals	HUC8	Phosphorus inputs and fluxes	2002, 2007, 2012
NETL_EIA_PlantWater	Modified EIA Thermoelectric Plant Water Withdrawals	Water	National	Water discharge, consumption, withdrawal	2015
NOAA_FisheryLandings	National Oceanic and Atmospheric Administration Fisheries	Money	State	Fishery landings	2012–2018
StatCan_GDP	Statistics Canada Gross Domestic Product	Money	Canada	GDP for Canada	2010–2015
StatCan_IWS_MI	Statistics Canada Industrial Water Survey	Water	Country	Water use by NAICS	2005, 2007, 2009, 2011, 2013, 2015
StatCan_LFS	Statistics Canada Labour Force Study	Employment	Canada	Employment by industry	2010–2019
USDA_ACUP_Fertilizer	Chemical Use Survey	Chemicals	State	Fertilizer use by crop	2012, 2015, 2017, 2018, 2020
USDA_ACUP_Pesticide	Chemical Use Survey	Chemicals	State	Pesticide use by crop	2012, 2015, 2017, 2018, 2020
USDA_CoA_Cropland	USDA Census of Agriculture	Land, Other	County	Crop area by farm size and irrigation status by crop	2012, 2017
USDA_CoA_Cropland_NAICS	USDA Census of Agriculture	Land	State	Crop area by farm size and irrigation status by NAICS	2012, 2017
USDA_CoA_Livestock	USDA Census of Agriculture	Other	County	Livestock count by farm size	2012, 2017
USDA_ERS_FIWS	USDA Farm Income and Wealth Statistics	Money	National, State	Cash receipts value	2010–2019
USDA_ERS_MLU	USDA Major Land Uses	Land	National	Land use by category	2007, 2012
USDA_IWMS	USDA Irrigation and Water Management Survey	Water	State	Water application rate by state and crop	2013, 2018
USGS_MYB	USGS Mineral Yearbook	Geological	National	Imports, Exports, Production, Consumption	2012–2018
USGS_NWIS_WU	US Geological Survey Water Use in the US	Water	County	Annual national-level water use by various activities	2010, 2015
USGS_SPARROW	USGS SPARROW MAPPERS	Chemicals	HUC	Phosphorus and nitrogen in streams and coastal waters	2012
USGS_WU_Coef	USDA Water Use Coefficients	Water	National	Method for estimating water withdrawals for livestock	2005

**Table 3.** Activity-to-Sector Mapping for USGS_NWIS_WU flow-by-activity.

Activity SourceName	Activity	SectorSourceName	Sector	SectorDescription	SectorType
USGS_NWIS_WU	Industrial	NAICS_2012_Code	1133	Logging	I
USGS_NWIS_WU	Industrial	NAICS_2012_Code	23	Construction	I
USGS_NWIS_WU	Industrial	NAICS_2012_Code	31–33	Manufacturing	I
USGS_NWIS_WU	Industrial	NAICS_2012_Code	48839	Other Support Activities for Water Transportation	I
USGS_NWIS_WU	Industrial	NAICS_2012_Code	5111	Newspaper, Periodical, Book, and Directory Publishers	I
USGS_NWIS_WU	Industrial	NAICS_2012_Code	51222	Integrated Record Production/Distribution	I
USGS_NWIS_WU	Industrial	NAICS_2012_Code	51223	Music Publishers	I
USGS_NWIS_WU	Industrial	NAICS_2012_Code	54171	Research and Development in the Physical, Engineering, and Life Sciences	I
USGS_NWIS_WU	Industrial	NAICS_2012_Code	56291	Remediation Services	I
USGS_NWIS_WU	Industrial	NAICS_2012_Code	81149	Other Personal and Household Goods Repair and Maintenance	I

**Table 4.** Flow-By-Sector methods available in FLOWSA v1.2.1.

Data	Method Name	Years	Geographic Scale	Available Methods
Commercial non-hazardous waste for construction	CNHWC_national_20XX	2014	National	1
Commercial non-hazardous waste	CNHW_CA_20XX	2014	California	1
Commercial non-hazardous waste	CNHW_national_20XX	2014	National	1
Commercial RCRA-defined hazardous waste	CRHW_national_20XX	2017	National	1
Commercial RCRA-defined hazardous waste	CRHW_state_20XX	2017	National	1
Criteria and hazardous air emissions	CAP_HAP_national_20XX	2017	National	1
Electricity generation emissions	Electricity_gen_emissions_national_20XX	2016	National	1
Employment	Employment_national_20XX	2017	National	1
Employment	Employment_state_20XX	2012–2017	State	1
Land use	Land_national_20XX	2012	National	1
Point source industrial releases to ground	GRDREL_national_20XX	2017	National	1
Point source industrial releases to ground	GRDREL_state_20XX	2017	State	1
Point source releases to water	TRI_DMR_national_20XX	2017	National	1
Point source releases to water	TRI_DMR_state_20XX	2017	State	1
Water withdrawal	Water_national_20XX	2010, 2015	National	3
Water withdrawal	Water_state_20XX	2015	State	1

**Table 5.** FLOWSA v1.2.1 code statistics.

Type	File Count	Lines Code

py	109	24,730
yaml	136	5220
csv	78	79,163

## Data Availability

FLOWSA is a Python package developed by the USEPA. The package is actively maintained and publicly available on GitHub at https://github.com/USEPA/flowsa (accessed on 2 May 2022). FLOWSA is designed to be used with Python 3.7 and higher. Many of the datasets output by FLOWSA are stored on the USEPA’s Data Commons at https://edap-ord-data-commons.s3.amazonaws.com/index.html?prefix=flowsa/ (accessed on 2 May 2022).
