# Supplementary material for: FLOWSA: A Python Package Attributing Resource Use, Waste, Emissions, and Other Flows to Industries
Source: Appl Sci (Basel). Author manuscript; Available in PMC 2022 Nov 2. (PMC9628186; doi:10.3390/app12115742)
Supplement: Supplementary Material [file NIHMS1841091-supplement-Supplementary_Material.pdf]

Supplementary Information for FLOWSA: A Python package attributing resource use, waste, emissions, and other flows to industries

Catherine Birney, U.S. Environmental Protection Agency, Office of Research and Development, Center for Environmental Solutions and Emergency Response
Ben Young, Eastern Research Group, Inc.
Mo Li, GDIT
Melissa Conner, GQC
Jacob Specht, GQC
Wesley W. Ingwersen, U.S. Environmental Protection Agency, Office of Research and Development, Center for Environmental Solutions and Emergency Response

The following tables describe in detail the information captured in Flow-By-Activity (FBA) and Flow-By-Sector (FBS) tables.

Flow-By-Activity Format Specs

Field	Type	Required?	Description
Class	String	Y	Class of the flow. See Flow Classes.
SourceName	String	Y	Name of data source
FlowName	String	Y	ID or name of flow in its native source
FlowAmount	Numeric	Y	The amount of a given flow in its native unit
Unit	String	Y	SI unit acronym. `kg` for mass flows; `MJ` for energy flows
FlowType	String	Y	`ELEMENTARY_FLOW`, `TECHNOSPHERE_FLOW`, or `WASTE_FLOW`. See http://greendelta.github.io/olca-schema/FlowType.html
ActivityProducedBy	String	N*	An activity defined by the source producing a flow.
ActivityConsumedBy	String	N*	An activity defined by the source receiving/consuming a flow.
Compartment	String	Y	Name of compartment to which release goes, e.g. “air,” “water,” “ground.”
Location	String	Y	A numeric representation of the activity location, at a national, state, or county level
LocationSystem	String	Y	Description and year of the Location code, generally FIPS or ISO, e.g. `FIPS_2015`

Year	Int	Y	Year of data, e.g. `2010`
MeasureofSpread	String	N	A measure of spread of a frequency distribution. Acceptable values are `RSD` for relative standard deviation (aka coefficient of variation) are `SD` for the normal (aka 'arithmatic') standard deviation, `GSD` for geometric standard deviation
Spread	Numeric	N	The value for the given measure of spread.
DistributionType	String	N	The form of the frequency distribution, if given. Acceptable values are 'NORMAL,' 'LOGNORMAL,' 'TRIANGULAR,' 'UNIFORM.'
Min	Numeric	N	The minimum FlowAmount, if provided for the data range.
Max	Numeric	N	The maximum FlowAmount, if provided for the data range.
DataReliability	Numeric	Y	A score of data reliability based on reporting values associated with the amount. See Data Quality Pedigree Matrix.
DataCollection	Numeric	Y	A score of data collection based on reporting values associated with the amount. See Data Quality Pedigree Matrix.
Description	String	Y	Original description of the flow

*At minimum, either ActivityProducedBy or ActivityConsumedBy must be present. If there is a transfer between activities, both must be present.

Flow-By-Sector Format Specs

Field	Type	Required?	Description
Flowable	String	Y	Name of the flow. See 'Flowable' in fedelemflowlist FlowList
Class	String	Y	Class of flow
FlowAmount	Numeric	Y	The amount of a flow. Uses metric reference units.
SectorProducedBy	String	N*	A valid code from the SectorSourceName system (e.g. '31' for 'NAICS_2012_Code')
SectorConsumedBy	String	N*	A valid code from the SectorSourceName system
SectorSourceName	String	Y	By default, `NAICS_2012_Code`. Must be the same for SectorProducedBy and SectorConsumedBy.
Context	String	Y	Full context for the flow, e.g. "air," "water," "ground."
Location	String	Y	A numeric representation of the activity location, at a national, state, or county level
LocationSystem	String	Y	Description and year of the Location code, generally FIPS or ISO, e.g. `FIPS_2015`
Unit	String	Y	SI unit acronym. 'kg' for mass flows; 'MJ' for energy flows.
FlowType	String	Y	`ELEMENTARY_FLOW`, `TECHNOSPHERE_FLOW`, or `WASTE_FLOW`. See http://greendelta.github.io/olca-schema/FlowType.html
Year	Int	Y	Year of data, e.g. `2010`
MeasureofSpread	String	N	A measure of spread of a frequency distribution. Acceptable values are `RSD` for relative standard deviation (aka coefficient of variation) are `SD` for the normal (aka 'arithmatic') standard deviation, `GSD` for geometric standard deviation
Spread	Numeric	N	The value for the given measure of spread.
DistributionType	String	N	The form of the frequency distribution, if given. Acceptable values are 'NORMAL,' 'LOGNORMAL,' 'TRIANGULAR,'

			'UNIFORM.'
Min	Numeric	N	The minimum FlowAmount, if provided for the data range.
Max	Numeric	N	The maximum FlowAmount, if provided for the data range.
DataReliability	Numeric	Y	A score of data reliability based on reporting values associated with the amount. See Data Quality Pedigree Matrix.
TemporalCorrelation	Numeric	Y	A 1-5 score of data collection based on reporting values associated with the amount. See Data Quality Pedigree Matrix.
GeographicalCorrelation	Numeric	Y	A 1-5 score of data collection based on reporting values associated with the amount. See Data Quality Pedigree Matrix.
TechnologicalCorrelation	Numeric	Y	A 1-5 score of data collection based on reporting values associated with the amount. See Data Quality Pedigree Matrix.
DataCollection	Numeric	Y	A 1-5 score of data collection based on reporting values associated with the amount. See Data Quality Pedigree Matrix.
MetaSources	String	Y	The major data source(s) value is based on, usually a FlowByActivity set.
FlowUUID	String	Y	UUID from Federal Commons Flow List.

- At minimum, either SectorProducedBy or SectorConsumedBy must be present. If there is a transfer between sectors, both must be present.
